# Respiratory infections during lithium and valproate medication: a within-individual prospective study of 50,000 patients with bipolar disorder

**DOI:** 10.1186/s40345-020-00208-y

**Published:** 2021-02-01

**Authors:** Mikael Landén, Henrik Larsson, Paul Lichtenstein, Johan Westin, Jie Song

**Affiliations:** 1 Section of Psychiatry, Institute of Neuroscience and Physiology, The Sahlgrenska Academy at University of Gothenburg, Blå Stråket 15, 413 45 Gothenburg, Sweden; 2 Department of Medical Epidemiology and Biostatistics, Karolinska Institutet, Stockholm, Sweden; 3 Department of Medical Sciences, Örebro University, Örebro, Sweden; 4 Department of Infectious Diseases, The Sahlgrenska Academy at University of Gothenburg, Gothenburg, Sweden

**Keywords:** Respiratory tract infections, Lithium, Drug repositioning, Viruses, Therapeutic use, Coronavirus

## Abstract

**Background:**

In vitro studies have demonstrated that lithium has antiviral properties, but evidence from human studies is scarce. Lithium is used as a mood stabilizer to treat patients with bipolar disorder. Here, the aim was to investigate the association between lithium use and the risk of respiratory infections in patients with bipolar disorder. To rule out the possibility that a potential association could be due to lithium’s effect on psychiatric symptoms, we also studied the effect of valproate, which is an alternative to lithium used to prevent mood episodes in bipolar disorder.

**Method:**

We followed 51,509 individuals diagnosed with bipolar disorder in the Swedish Patient register 2005–2013. We applied a within-individual design using stratified Cox regression to estimate the hazard ratios (HRs) of respiratory infections during treated periods compared with untreated periods.

**Results:**

During follow-up, 5,760 respiratory infections were documented in the Swedish Patient Register. The incidence rate was 28% lower during lithium treatment (HR 0.73, 95% CI 0.61–0.86) and 35% higher during valproate treatment (HR 1.35, 95% CI 1.06–1.73) compared with periods off treatment.

**Conclusions:**

This study provides real-world evidence that lithium is associated with decreased risk for respiratory infections and suggests that the repurposing potential of lithium for potential antiviral or antibacterial effects is worthy of investigation.

## Introduction

The outbreak of the coronavirus SARS-CoV-2, the cause of COVID-19 (Corona virus Disease-2019) respiratory disease, has prompted a search for approved drugs with antiviral properties that might be repurposed to treat covid-19, or shed light on biological mechanisms that might be targeted for antiviral purposes.

Lithium has been suggested to have antiviral properties since the 1970-1980s (Rybakowski 2000; van den Ameele et al. 2016; Murru et al. 2020). In vitro studies have shown that lithium inhibit replication of several viruses (Skinner et al. 1980; Ren et al. 2011; Chen et al. 2016; Zhao et al. 2017; Qian et al. 2018). Interestingly, in vitro studies have also shown lithium to effectively suppress infection with Porcine deltacoronavirus (Zhai et al. 2019) and other viruses that belong to the Coronaviridae family (Murru et al. 2020), albeit usually in concentrations that would be toxic in humans (Nowak and Walkowiak 2020).

As pointed out in a recent review (Murru et al. 2020), however, there is scarce evidence to support that lithium therapy affects the risk of infections or infection severity in humans. Small studies in the 1990s suggested reduced rates of herpes virus infections with lithium (Amsterdam et al. 1990a, b; Amsterdam et al. 1990a, b; Amsterdam et al. 1996). A retrospective review of 236 patients with affective disorders found that lithium treated patients showed a small reduction of flu-like illness during lithium therapy (Amsterdam et al. 1998).

Lithium is the mainstay prophylactic treatment of bipolar disorder (Joas et al. 2017). Here, we leveraged a Swedish cohort of more than 50,000 patients with bipolar disorder followed over eight years to investigate if lithium treatment is associated with risk of respiratory infections. We employed a within-individual design to circumvent the problem of confounding by indication. With each person serving as his or her own matched control, the within-individual design controls for fixed confounders (e.g., baseline severity of the illness, genetic predisposition, childhood environment) (Lichtenstein et al. 2012). To rule out the possibility that a potential association between lithium and respiratory infection rate is secondary to the effect on psychiatric symptoms, we also calculated the association between valproate medication and respiratory infections. Valproate is a commonly used alternative to lithium to prevent mood episodes in bipolar disorder (Cipriani et al. 2013), recommended by the National Institute for Health and Care Excellence (NICE) when lithium is not suitable (Kendall et al. 2014).

## Methods

### Subjects

We linked several longitudinal Swedish population-based registers through unique personal identification numbers (Ludvigsson et al. 2009): the Total Population Register, the Migration Register, the Cause of Death Register, the Patient Register (captures information on inpatient care since 1973 and outpatient visits to specialists care since 2001), and the Prescribed Drug Register (Wettermark et al. 2007). We identified 51,509 individuals with bipolar disorder followed from 1 October 2005, or age 15, or date of bipolar disorder diagnosis (if later than 1 October 2005) until emigration, death, or 31 December 2013, whichever occurred first. This date was chosen because the register linkage was conducted during 2015 with access to data up to December 2013. This study was approved by the Ethics Committee at Karolinska Institutet.

### Bipolar disorder cases

To identify cases with bipolar disorder in the Patient Register, we applied a validated algorithm (Sellgren et al. 2011) that has been used in several previous Swedish register-based studies on bipolar disorder (Viktorin et al. 2014, 2017; Song et al. 2017). This algorithm includes bipolar I disorder, bipolar II disorder, bipolar disorder not otherwise specified, or schizoaffective disorder bipolar type.

### Medications

The main exposure was defined as dispensed prescriptions of lithium sulphate (Anatomical Therapeutic Chemical classification code: N05AN01) or sodium valproate/valproic acid (N03AG01) as documented in the prescribed drug register. In Sweden, long-term prescribed medications are dispensed for three-month periods. Similar to previous studies (Song et al. 2017), we therefore defined a medication period as a sequence of at least two prescriptions, with no more than three months (92 days) between any two consecutive prescriptions. Thus, for both lithium and valproate, individuals were defined as on medication during the time interval between two dispensed prescriptions, unless the dispensed prescriptions occurred more than three months apart. To determine whether an individual was on/off medication initially, the start of follow-up was set to 1 October 2005 because the prescribed drug register started 1 July 2005.

### Outcomes

In Sweden, uncomplicated respiratory infections are commonly treated in primary health care and therefore not recorded in the Patient register. Viral infections complicated by bacterial superinfection may, however, warrant specialist care or warrant hospital in-patient treatment. Hence, patients with a viral respiratory infection are likely to be diagnosed with bacterial pneumonia. A systematic review and meta-analysis of 27 studies, including a total of 3,215 patients, found that bacterial coinfection was very common (up to 65%) among patients hospitalized for influenza (Klein et al. 2016). The authors conclude that bacterial coinfection should always be suspected in all patients with influenza. We therefore defined outcome as being diagnosed with either viral or bacterial respiratory infections. Specifically, the outcomes were defined as influenza or other viral pneumonia (ICD-10 codes: J09, J10, J11.0, J11.1, J12), bacterial pneumonia (ICD-10 codes: J13, J14, J15, J16), pneumonia in other diseases (ICD-10 code: J17.1), pneumonia due to unspecified organisms (ICD-10 code: J18), acute bronchitis (ICD-10 code: J20), acute bronchiolitis (ICD-10 code: J21), and unspecified acute lower respiratory infection (ICD-10 code: J22). In an additional posthoc analysis, we limited outcomes to respiratory infection diagnoses specifically caused by viruses (ICD-10 codes: J09, J10, J11.0, J11.1, J12, J17.1, J20.3-7, and J21.0-1). Dates and diagnoses were retrieved from the National Patient Register that covers in-patient care and doctor visits from private and public caregivers, with the exception of primary care which is not covered.

### Statistical analyses

We performed within-individual analyses using stratified Cox regression. By comparing treated and untreated periods within the same individual, this approach automatically controls for all time-stationary confounders and thus reduces the likelihood of confounding by indication. More details about this method are given in a review (Allison 2009).

We split the follow-up time into consecutive periods. A new period started after a medication switch (i.e., from off medication to on medication or vice versa, for either lithium or valproate) or a respiratory infection diagnosis. For the latter, we restarted the following period at baseline (i.e., to set the underlying time scale as the time since last diagnosis). Lithium and valproate treatment status were defined as time-varying dichotomous exposures. Age band (15–51, 51–64, 64–75, 75–100 years, grouped by the quartiles of the baseline age distribution among those who had an event during the follow-up) was adjusted for as a time-varying categorical covariate. We estimated the hazard ratios (HRs) and 95% confidence intervals (CIs) for respiratory infections during periods with treatment as compared with periods off treatment. This method have previously been described in detail (Lichtenstein et al. 2012; Chang et al. 2014; Chen et al. 2014; Molero et al. 2015).

All analyses were performed with Stata 16.0 (StataCorp. 2013).

## Results

Among the 51,509 patients with bipolar disorder, a total of 5760 respiratory infections were diagnosed in 3,389 individuals (6.6%) during 217,214 person-years of follow-up (Table 1). Lithium treatment was more prevalent (41.0%) than valproate treatment (15.9%). About 50% patients were not exposed to lithium or valproate during the study period. Additional file 1: Table S1 outlines the characteristics of patients treated with lithium, valproate, or both during the study period.

**Table 1:** Characteristics at baseline and during follow-up for patients with bipolar disorder with different medication status in Sweden 2005–2013

	Lithium^a^	Valproate^a^	Never treated with lithium/valproate	Total	Eligible for within-individual analysis^c^
Number of individuals (%)	21,126 (41.0)	8410 (15.9)	25,758 (49.7)	51,509	3285 (6.4)
Sex (% male)	8469 (40.1)	3609 (42.9)	9001 (34.9)	19,475 (37.8)	1307 (39.8)
Characteristics at baseline					
Age distribution (N of individuals, %)^b^					
15–51	11,355 (53.7)	5115 (60.8)	16,263 (63.1)	30,808 (59.8)	864 (26.3)
51–64	5513 (26.1)	1998 (23.8)	4986 (19.4)	11,494 (22.3)	899 (27.4)
64–75	2652 (12.6)	887 (10.5)	2498 (9.7)	5496 (10.7)	798 (24.3)
75–100	1606 (7.6)	410 (4.9)	2011 (7.8)	3711 (7.2)	724 (22.0)
Respiratory infection (N of individuals, %)					
At least one respiratory infection event during follow-up	1532 (7.3)	722 (8.6)	1463 (5.7)	3389 (6.6)	3285 (100)
Proportion of time exposed to lithium or valproate medications	41.3	42.3	–	22.4	28.6

^a^3785 individuals were ever treated with both lithium and valproate (defined as at least 3 months treatment)

^b^Age band was categorized according to the quartiles of age distribution of individuals who ever have any respiratory infection at any time during follow-up

^c^Includes individuals who ever had a respiratory infection during the follow-up; individuals without any of the outcome diagnoses during follow-up do not provide information

After excluding individuals without respiratory infections and varying covariates (i.e., treatment status and age band) during follow-up, 3285 individuals were eligible for the within-individual analysis. These patients experienced a total of 5656 respiratory infections.

The rate of respiratory infection diagnoses was 27% lower during periods on lithium treatment compared with periods off lithium (HR 0.73, 95% CI 0.61–0.86). By contrast, the rate of respiratory infections was higher during periods with valproate treatment (HR 1.35, 95% CI 1.06–1.73).

When we in a post hoc analysis limited the diagnoses to viral respiratory infections, the number of events dropped by 97% to 199. In this analysis, neither lithium (HR 1.07, 95% CI 0.35–3.22) nor valproate (HR 0.96, 95% CI 0.24–4.37) was significantly associated with the risk of viral respiratory infections.

## Discussion

In a large sample of patients with bipolar disorder, we found that rates of respiratory infection diagnosed at hospitals or specialist outpatient care were significantly lower while on lithium medication than during periods off lithium. By contrast, the rate of respiratory infections was increased during medication with the alternative mood stabilizer valproate. Given that we used a within-individual design—and that lithium and valproate both effectively prevent mood episodes in bipolar disorder—the difference is likely to be caused by effects of the medication used.

Overall, the proportion of respiratory infections was higher in patients treated with lithium (7.3%) and valproate (8.6%) than in patients who were treated with any of these drugs (5.7%). However, this difference is likely to be confounded by indication, which is why a between group comparison does not provide information on whether lithium or valproate influence the risk of respiratory infections. For example, cases treated with lithium or valproate are more likely to have a more severe psychiatric disorder, to smoke, or have other unknown risk factors than cases never treated with any of these drugs. Moreover, cases that had used lithium or valproate were older and were more likely to be male than those who had never used lithium or valproate. The within-individual model that we used compares the risk of respiratory infections during periods with lithium (or valproate) medication with the risk during periods without lithium (or valproate) medication *within each individual*. The model thereby corrects for potential group differences such as sex, genetical make up, severity of the psychiatric disorder et cetera.

Bacterial coinfections or superinfections is a common complication of viral respiratory infections, especially in hospitalized patients (Klein et al. 2016). In the present study, it was not possible to distinguish between viral and bacterial respiratory infections because the results are compatible with effects on both viral and bacterial infections. The observed association of lithium treatment and low risk of respiratory infections therefore needs to be addressed in further investigations using broad diagnostic panels for viral as well as bacterial pathogens. The previously reported antiviral properties of lithium might, however, explain our results since an antiviral mechanism may also protect against bacterial complications.

Limited testing for viral pathogens in clinical routine care may explain the dramatic decrease of number of events (97%) when we limited the analysis to viral respiratory only infections in a post hoc analysis. This drop in sample size radically impaired statistical power and the post hoc analysis did not yield any significant results.

Although the antiviral effect of lithium remains speculative in vivo, our findings are in line with a small previous study (N = 236) that found a reduction of flu-like illness during lithium therapy.(Amsterdam et al. 1998) Although lithium is believed to act across several biological pathways and the exact mechanism of actions remains obscure (Alda 2015), it is known to inhibit glycogen synthase kinase 3β (GSK3β) (Malhi and Outhred 2016). GSK3β has been suggested to be involved in inducing apoptosis and release of viral particles in dengue virus-2 infection (Cuartas-Lopez and Gallego-Gomez 2020), and GSK3β suppression is hence one potential mechanism underlying the antiviral effect of lithium (Sarhan et al. 2017). Lithium has also been found to induce leucocytosis (Carmen et al. 1993; Petrini and Azzara 2012), and attenuate anti-inflammatory effects during chronic use (reviewed by (van den Ameele et al. 2016)). Further, lithium may stimulate immunoglobulin production in B-lymphocytes, increase T-lymphocyte proliferation, and affect proinflammatory cytokine production (reviewed by (Maddu and Raghavendra 2015) and (van den Ameele et al. 2016)).

Valproate treatment, by contrast, *increased* the risk of respiratory infections. It is well known that valproate can cause reversible bone marrow suppression leading to leukopenia and neutropenia (Acharya and Bussel 2000). However, a meta-analysis of infectious diseases reported as adverse events during the double-blind phase of placebo-controlled randomized clinical studies failed to show a significant effect of valproate (Zaccara et al. 2017). It should be noted, however, that groups treated with lithium or valproate are likely to differ in several respects (Additional file 1: Table S1). Lithium is the recommended first treatment option in Sweden, why the group treated with valproate is likely to be less responsive to treatment and might also be a more severely ill group.

This is by far the largest study testing the hypothesis that lithium protects against respiratory infections. The within-individual design controls for time-invariant covariates that might confound the results, but the method is susceptible to confounding by factors associated with the exposure that change during the observation period. For example, periods on a mood stabilizer might be associated with better access to healthcare than periods off a mood stabilizer. Although we controlled for this by including valproate in our analyses, it cannot be excluded that time-varying confounding might differ between lithium and valproate. The main limitation is that we cannot discern if lithium protects against viral, bacterial, or other aetiologies. Another limitation is that we lack information on tobacco use and comorbidity, but this should not confound the results in a within-individual model. Limitations finally include the lack of information regarding adherence, but this would if anything be a conservative bias.

In conclusion, we provide real-world evidence that lithium treatment reduces the rate of respiratory infections. Taken together with ample previous literature on antiviral effects of lithium (Murru et al. 2020), the repurposing potential of lithium for antiviral effects is worth pursuing.

## Supplementary Information

**Additional file 1: Table S1.** Characteristics of individuals with bipolar disorder by different treatment.

## Data Availability

The authors had full and ongoing access to the original data presented and analysed in this study. Due to Swedish legal restrictions, register data cannot be shared. However, the data that support the findings of this study are available from the corresponding author upon reasonable request.
