# Supplementary material for: Respiratory infections during lithium and valproate medication: a within-individual prospective study of 50,000 patients with bipolar disorder
Source: Int J Bipolar Disord. 2021 Feb 1;9:4. doi: 10.1186/s40345-020-00208-y (PMC7847747; doi:10.1186/s40345-020-00208-y)
Supplement: Supplementary file 1 — Additional file 1: Table S1. Characteristics of individuals with bipolar disorder by different treatment. [file 40345_2020_208_MOESM1_ESM.pdf]

Additional file 1:

Table S1. Characteristics of individuals with bipolar disorder by different treatment

Title

Respiratory infections during lithium and valproate medication: a within-individual prospective study of 50,000 patients with bipolar disorder

Authors

Mikael Landén, M.D., Ph.D., Henrik Larsson, Ph.D., Paul Lichtenstein, Ph.D., Johan Westin, M.D., Ph.D., Jie Song, Ph.D.

Correspondence

Mikael Landén, Section of psychiatry, Sahlgrenska University hospital, Blå Stråket 15, 413 45 Gothenburg, Sweden. E-mail: mikael.landen@gu.se

Table 1. Characteristics of individuals with bipolar disorder by different treatment

	Lithium only	Valproate only	Both lithium and valproate[c]
N	17,777	5,925	6,178
Average birth year (SD)	1958 (18.8)	1964 (17.8)	1964 (16.4)
Sex (% male)	39.3	42.6	41.4
Psychiatric comorbidities			
ADHD[a] (%)	8.2	18.3	15.8
Eating disorders (%)	2.8	4.1	4.2
ASD[a] (%)	2.6	5.5	4.5
Anxiety disorders (%)	34.7	47.1	44.5
Personality disorders (%)	14.8	23.8	23.3
Alcohol abuse (%)	15.8	25.2	23.6
Drug abuse (%)	13.1	22.6	23.0
Suicide-related events (%)	16.5	20.9	25.3
Concomitant psychotropic treatment[b]			
Antidepressants (%)	75.3	83.2	83.2
Anticonvulsants (%)	23.0	31.7	39.2
Antipsychotics (%)	67.2	75.5	89.3
Lamotrigine (%)	38.3	41.7	53.2
Benzodiazepines (%)	55.6	59.8	70.4

a) Abbreviations: ADHD, attention-deficit/hyperactivity disorder; ASD, autism spectrum disorders. b) Drugs are defined with Anatomical Therapeutic Chemical (ATC) classification codes: lamotrigine (N03AX09), antipsychotics (N05A) excluding lithium, antidepressants (N06A), anticonvulsants (N03A) excluding lamotrigine and valproate and benzodiazepines (N05BA). c) Individuals with both lithium and valproate were those who were ever treated with lithium and valproate during the study period, not necessarily at the same time.
